# The Roles of lncRNA in Myocardial Infarction: Molecular Mechanisms, Diagnosis Biomarkers, and Therapeutic Perspectives

**DOI:** 10.3389/fcell.2021.680713

**Published:** 2021-09-16

**Authors:** Luhan Xie, Qingqing Zhang, Jun Mao, Jun Zhang, Lianhong Li

**Affiliations:** ^1^Department of Pathology and Forensic Medicine, College of Basic Medical Sciences, Dalian Medical University, Dalian, China; ^2^Department of Teaching Affairs, Dalian Medical University, Dalian, China

**Keywords:** myocardial infarction, long non-coding RNAs, ceRNA, exosome, biomarker

## Abstract

In recent years, long non-coding RNAs (lncRNAs) have been demonstrated to be associated with many physiological and pathological processes in cardiac. Recent studies have shown that lncRNAs are expressed dynamically in cardiovascular diseases and participate in regulation through a variety of molecular mechanisms, which have become a critical part of the epigenetic and transcriptional regulatory pathways in heart development, as well as the initiation and progress of myocardial infarction. In this review, we summarized some current research about the roles of lncRNAs in heart development and myocardial infarction, with the emphasis on molecular mechanisms of pathological responses, and highlighted their functions in the secondary changes of myocardial infarction. We also discussed the possibility of lncRNAs as novel diagnostic biomarkers and potential therapeutic targets for myocardial infarction.

## Introduction

According to the latest report of the World Health Organization (WHO), cardiovascular diseases are still the leading cause of death worldwide, and coronary heart disease (CHD) accounts for the largest proportion. Myocardial infarction (MI) is a serious manifestation of CHD, which results from acute and prolonged deficits in the supply of oxygen, nutrients, and survival factors to the myocardium, trigger a series of severe biochemical and metabolic perturbations in the cardiomyocyte. This imbalance is most caused by sudden and prolonged ischemia. In contrast to other cardiac diseases, where multiple processes contribute to pathogenesis, cell death in the ischemic area is the initial and central event of myocardial infarction. Besides, infarction is associated with a variety of structural and functional consequences, and the most irreversible of which is the death of cardiomyocytes (CMs) (Del Re et al., 2019). However, cardiomyocytes are terminally differentiated cells and have lost their mitotic ability (Mercola et al., 2011), so the regeneration capacity of the heart in adult mammals is limited. Meanwhile, infarct size is important as a major determinant of subsequent cardiac dysfunction and mortality (Del Re et al., 2019).

For a long time, proteins have been regarded as the center of the gene regulatory networks. Nevertheless, with the development of new techniques, only 2% of protein-coding genes were found in the mammalian genome (Shabalina and Spiridonov, 2004), whereas the vast majority of the human genome is transcribed into non-coding RNAs, including long non-coding RNAs (lncRNAs). Genome-wide association studies have shown that a significant portion of the genetic variation related to human disease resides in the non-coding regions of the genome (Partridge et al., 2020; Van Nostrand et al., 2020). To date, ncRNAs with gene regulatory functions have been identified and shown to play a role in various biological processes, including epigenetic and transcriptional regulation (Rinn and Chang, 2012), embryogenesis and development (Pauli et al., 2011), pluripotency and differentiation (Guttman et al., 2011; Kretz et al., 2013), protein biosynthesizing process, as well as participate in dynamic developmental and cell-specific expression patterns.

The expression of lncRNA in different tissues is more specific than protein-coding genes, indicating the function of lncRNAs is closely related to the functional specificity of tissues (Ransohoff et al., 2018). Besides, most lncRNAs showed tissue-specific expression patterns similar to master transcription factors. A recent study characterized cardiac-enriched lncRNAs. By comparing RNA sequencing data from mouse hearts, livers, and skin cells, the study identified 321 cardiac-expressed lncRNAs. Among them, 117 transcripts were abundant and highly cardiac enriched. 67% of the cardiac-enriched lncRNAs were also highly expressed in isolated cardiomyocytes, indicating that the majority of the highly expressed lncRNAs in the mouse hearts are expressed by cardiomyocytes (Matkovich et al., 2014). Therefore, lncRNAs may represent important regulatory factors of development and disease processes in hearts, as well as novel disease biomarkers or therapeutic targets for cardiovascular disease and its risk factors. A deeper understanding of the functional and molecular mechanisms of lncRNAs will have profound implications for cardiovascular disease and may provide new opportunities for intervention in disease progression.

In this review, we summarized some of the studies on the critical roles of lncRNAs in many different aspects of cardiac physiology and pathology, especially in myocardial infarction, as well as discussed the possibility of lncRNAs as novel diagnostic biomarkers and therapeutic targets for myocardial infarction. We also discussed the existing challenges and future trends in the clinical practice and lab research of lncRNAs in MI.

## lncRNAs and Roles in Cardiac Development and Differentiation

lncRNAs are defined as non-coding RNA sequences greater than 200 nt in length, some lncRNAs can produce small peptides, but the vast majority of lncRNAs do not encode proteins. According to the NON-CODE database^1^, the number of lncRNAs is 549,813 in total, including 173,112 and 131,974 lncRNA genes for human and mice, respectively, and many lncRNAs appear to be cardiac-specific, or cardiac-enriched (Grote et al., 2013; Klattenhoff et al., 2013; Matkovich et al., 2014; Ounzain et al., 2015).

lncRNA is an important part of the gene regulatory network for embryonic stem cell differentiation, alteration of its expression can interfere with the maintenance and differentiation (Guttman et al., 2011). Changes in transcriptional networks during heart development and homeostasis may lead to congenital heart disease and chronic heart problems (Srivastava, 2006; Bruneau, 2008). A genome-wide transcriptional profiling study confirmed that lncRNAs showed significant stage-specific expression manner in cardiac differentiation and abundant genes related to development, morphogenesis, and transcription. Moreover, the expression of lncRNAs is correlated with adjacent genes, suggesting that lncRNAs play a *cis-*regulatory role in cardiomyocyte differentiation and cardiac development (Wamstad et al., 2012).

The *Mesp1* gene (post mesoderm gene 1) is one of the earliest genes expressed in heart cells which is essential for heart development (Lescroart et al., 2014). AK143260, named Braveheart (*Bvht*), a mouse-specific lncRNA that is highly expressed in mouse embryonic stem cells (ESCs) and cardiomyocytes. During ESCs differentiation, *Bvht* acts as upstream of *Mesp1*, regulating the transformation of neonatal mesoderm to cardiac development (Klattenhoff et al., 2013). Moreover, during cardiomyocyte differentiation, *Bvht* mediates cardiac epigenetic regulation in cardiovascular core gene networks by activating PRC2 component SUZ12 and transcription factors. Lateral mesoderm-specific lncRNA *Fendrr* is also essential for normal heart and body wall development during tissue differentiation in mice embryos. *Fendrr* deficiency shows myocardial dysfunction, affects the development of the heart and body walls and contributes to embryonic death. Besides, *Fendrr* deletion affects the modification of epigenetic promoters and the expression of transcriptional regulators (Grote et al., 2013). It has also been suggested that *Fendrr* may need to balance H3K4 and H3K27 methylation during mesodermal differentiation to coordinate genetic programming (Rizki and Boyer, 2015).

The transition from the pluripotent stage to the differentiated stage is accompanied by a large number of epigenetic changes (Beermann et al., 2016). Most of the lncRNAs detected in human left ventricle samples contain DNaseI hypersensitivity loci and epigenetic markers of active signal transcription, indicating that cardiac lncRNA expression is correlated with epigenetic modification (Yang et al., 2014). Many novel lncRNAs with potential roles in cardio-genesis are also differentially regulated in human heart diseases (Ounzain et al., 2015). Therefore, lncRNAs are critical components in cardiac development, and participate in cardiac transcription, and serve as epigenetic regulators in cardiac gene expression.

## lncRNAs and Their Molecular Mechanism During MI

Previous studies have shown that, compared with the control group, multiple transcriptome changes occurred in the process of MI, with the largest transcriptome changes occurring in the acute phase (day 1) (Zhang X. et al., 2021), and the largest expression of lncRNA also occurred in the day 1 of MI. Meanwhile, these dysregulated lncRNAs are associated with the initiation and progression pathway of MI (Shi et al., 2020). Inflammation and cardiomyocyte apoptosis are the main characteristics of myocardial infarction (Marchant et al., 2012). After the initial ischemic event, the acute sudden death of cardiomyocytes in the infarcted zone rapidly activates the innate immune pathway, thereby trigger a strong inflammatory response (Frangogiannis, 2014). In the murine study, mice were sacrificed on the first-day post-MI, and differentially expressed lncRNA in infarcted heart tissue has a strong relationship with the inflammation-related pathways (Liu et al., 2014; Zangrando et al., 2014). These results suggest that lncRNA may participate in the regulation of MI in the acute phase through the inflammatory response.

**FIGURE 1:**
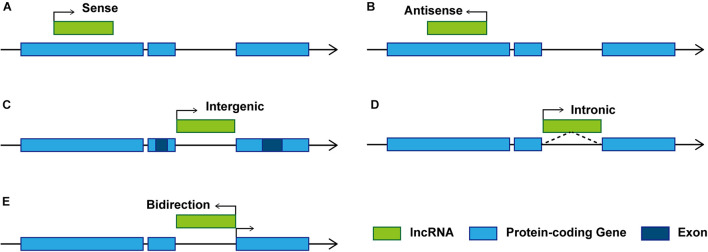
Schematic diagram of representative lncRNA categories. Based on the location of lncRNAs relative to adjacent protein-coding genes, lncRNAs can be classified as: **(A)** Sense. Transcribed from the sense strand of protein-coding genes, can be considered as transcription variants of protein-coding mRNAs, which usually overlap exons by sharing the same promoter (Ma et al., 2013). **(B)** Antisense. Transcribed from the antisense strand of protein-coding genes, overlapping with protein-coding genes or covering the entire protein-coding sequence through intron. **(C)** Intergenic. Also known as long intergenic non-coding RNAs (lincRNAs), transcribed between two protein-coding genes, and located distant from other genes (Schmitz et al., 2016). **(D)** Intronic. Transcribed from introns of protein-coding genes in either sense or antisense orientation. **(E)** Bidirectional. Transcribed in the opposite direction of the protein-coding gene promotor within 1 kb.

**FIGURE 2:**
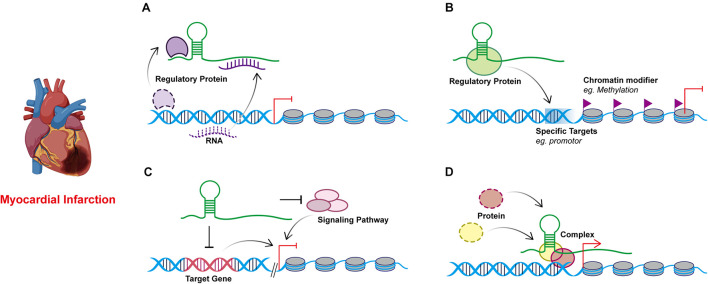
Models of four known lncRNA molecular mechanisms of action in myocardial infarction. **(A)** Decoy. lncRNAs can act as sponges to interact with regulatory proteins or RNAs away from their targets. **(B)** Guide. lncRNAs can recruit and combine regulatory proteins or DNAs through molecular interactions and guiding the complexes to the specific targets. **(C)** Signal. lncRNAs respond to specific stimuli and participant in the regulation of gene expression by regulating signaling pathways through *cis-* or *trans-* way. **(D)** Scaffold. lncRNAs may serve as scaffolds to bring proteins together in biological regulation.

Studies on the effect of lncRNAs have shown that lncRNAs regulate gene expression at both transcriptional and post-transcriptional levels. The complementary sites on lncRNA enable them to recognize and bind to mRNAs, miRNAs, or other lncRNAs and conduct highly specific regulation. Protein binding sites allow them to interact with proteins to produce various ribonucleoprotein particles with different biological functions (Thum and Condorelli, 2015). The function of lncRNA is related to its structure and genome localization. Based on their genomic organization and structure, lncRNAs can be categorized as being sense, antisense, intergenic, intronic, and bidirectional (Ponting et al., 2009; Figure 1) in contrast to miRNAs, lncRNAs have a diversity of molecular functioning classed as being signal, decoy, guide, or scaffold (Devaux et al., 2015; Figure 2 and Table 1).

**TABLE 1:** LncRNAs with characterized functions and target genes in myocardial infarction.

LncRNA	Target	Function	References
CARL	miR-539/PHB2	Inhibit mitochondrial fission and myocardial apoptosis in MI.	Wang et al., 2014
APF	miR-188-3p/ATG7	Regulates autophagic program and autophagic cell death after MI.	Wang K. et al., 2015
NRF	miR-873/RIPK1-RIPK3	Regulate cardiomyocyte necrosis and myocardial injury in I/R.	Wang et al., 2016
H19	miR-103/107/FADD	Regulate programmed Necrosis and Myocardial I/R Injury	Wang J.X. et al., 2015
	miR-877-3p/Bcl-2	Inhibit the mitochondrial apoptotic pathway in myocardial	Li et al., 2019
	miR-675/PPARα	Regulate cell death and IR injury	Luo et al., 2019
CAIF	p53-mediated myocardin	Repress autophagic cell death and alleviate MI.	Liu et al., 2018
*Kcnq1ot1*	RUNX3	Recruits DNMT1 to the RUNX3 promoter region and regulates CMEC viability and inflammatory response during MI.	Wang et al., 2019
CPR	MCM3	Interact and recruit DNMT3A to the CpG island of MCM3 promoter, then inhibit cardiomyocyte proliferation and cardiac function after MI	Ponnusamy et al., 2019
*Sarrah*	NRF2	Recruit CRIP2 and p300 to form complex and regulate cardiomyocytes apoptosis in AMI.	Trembinski et al., 2020
FAF	FGF9/FGFR2/PI3K/Akt	Regulate myocardial cell apoptosis in AMI.	Shi et al., 2019
SLC8A1-AS1	SLC8A1/cGMP-PKG	Reduce infarct size and ischemia damage in AMI.	Guo et al., 2019
Airn	Igf2bp2	Affects the translation of Igf2bp2, silencing Airn can increase apoptosis and affect the physiological function of cardiomyocytes.	Hosen et al., 2018
ZFAS1	SERCA2a	Inhibitor of SERCA2a and limits systolic function during MI.	Zhang et al., 2018
Ahit	SUZ12	Downregulating the expression of MEF2A and preventing cardiac hypertrophy through epigenomic modulation.	Yu J. et al., 2020
ANRIL	WDR5-HDAC3	Form protein complexes and increase ROS level.	Zhang et al., 2020

### Decoy

An increasing number of ceRNAs have emerged that regulate numerous processes of myocardial infarction through miRNA inhibition. miRNAs inhibit the translation or promote the degradation of mRNAs by binding to the complementary sequence of their 3′ untranslated region (3′-UTR), thus regulating gene expression, while lncRNAs can regulate the expression of mRNAs by acting as miRNAs sponges to interact with miRNAs (Cesana et al., 2011). The lncRNA-miRNA-mRNA axis can associate lncRNAs with the various regulatory functions in myocardial infarction.

lncRNA AK017121, named cardiac apoptosis-related lncRNA (CARL), acting as ceRNA in MI. PHB2 inhibits mitochondrial fission and apoptosis, miR-539 targeting PHB2 and subsequently up-regulating the level of PHB2 to inhibited mitochondrial fission and apoptosis. Meanwhile, the overexpression of CARL inhibiting the expression of miR-539. Modulation of the CARL-miR-539-PHB2 axis may provide a novel approach for the treatment of apoptosis and myocardial infarction (Wang et al., 2014). The lncRNA AK079427, named autophagy promoting factor (APF), can binds with miR-188-3p as a ceRNA and lead to increased ATG7 levels, which enhances cardiac autophagy in cardiomyocytes after MI. Indicating that APF may be viewed as a biomarker target in MI, and APF-miR-188-3p-ATG7 is a regulated axis of myocardial cell autophagy (Wang K. et al., 2015).

In addition to cell apoptosis and autophagy, ceRNA has also been shown to regulate necrosis in myocardial infarction. A long non-coding RNA, named necrosis-related factor (NRF), was closely related to necrotic death of cardiomyocytes by acting as an endogenous RNA sponge that interacted with miR-873. Experiments show that RIPK1 and RIPK3 participate in H_2_O_2_-induced cardiomyocytes necrosis, miR-873 suppresses the translation of RIPK1/RIPK3 and inhibits RIPK1/RIPK3-mediated necrotic cell death in cardiomyocytes, which reduces myocardial infarct size upon ischemia/reperfusion (I/R) injury in the animal model. Indicating NRF is transcriptionally activated by p53 upon I/R injury, binds miR-873, leading to increased cardiomyocyte necrosis mediated by RIPK1/RIPK3 (Wang et al., 2016). Another study showed that RIPK1/RIPK3-mediated cardiomyocyte necrosis can also be regulated by lncRNA H19. Fas-associated protein with death domain (FADD) influencing the formation of RIPK1/RIPK3 complexes in H_2_O_2_-treated H9C2 cells and MI mice, while H19 attenuates necrotic responses by targeting miR-103/107 and FADD (Wang J.X. et al., 2015). H19 was not only involved in myocardial cell necrosis, but also participated in the regulation of myocardial cell apoptosis. As a competitive endogenous RNA of miR-877-3p (Li et al., 2019) and miR-675 (Luo et al., 2019), H19 can regulate apoptosis of cardiomyocytes by regulating mitochondrial apoptosis pathway and targeting PPARα, respectively.

Moreover, lncRNAs can also bind transcription factors, thereby inhibiting their binding to genes. A lncRNA called cardiac autophagy inhibitory factor (CAIF) inhibiting the autophagy of cardiac cells during myocardial infarction by directly interacting with the transcription factor p53, then blocking its binding to the myocardin promoter region (Liu et al., 2018). Recent findings have shown that lncRNAs can also compete with miRNAs and interact with target mRNAs to inhibit gene expression. In some cases, the stability and function of lncRNAs can be reduced by regulating specific miRNAs. Besides, some lncRNAs can produce miRNAs themselves that inhibit target mRNAs (Yoon et al., 2014).

### Guide

In addition to mediating nucleic acid interactions, lncRNAs can also act as guide RNAs, forming complexes with proteins or DNAs through molecular interactions, acting on adjacent or distal genes in a *cis-* or *trans-* way, and guiding the complexes to locate specific target genes to regulate various biological phenomena. Cell characteristics in organisms are determined by epigenetic factors that regulate specific gene expression programs. These epigenetic factors, such as chromatin modification complexes and DNA methyltransferases, activate and inhibit specific genes by enzyme modifying chromatin and DNA (Moran et al., 2012).

For example, lncRNAs can modify transcription by recruiting chromatin modifiers and transcription factors to genomic sites to regulate gene expression (Ng et al., 2012; Grote et al., 2013). In the placenta, lncRNA *Kcnq1ot1* interacts with specific transcriptional repressors by recruiting chromatin remodeling complexes to establish a lineage-specific transcriptional silencing pattern (Pandey et al., 2008). In cardiac, *Kcnq1ot1* recruits DNA methyltransferase 1 (DNMT1) to the RUNX3 promoter region and inhibit Runx3 expression, regulating the proliferation and apoptosis of CMEC and induces inflammatory response during myocardial infarction (Wang et al., 2019). MCM3 (minichromosomal maintenance 3) is the initiator of DNA replication and cell cycle process, which can inhibit cardiomyocyte proliferation. lncRNA CPR represses MCM3 expression by directly interacting and recruiting DNMT3A to the CpG island of the MCM3 promoter, thereby preventing cardiomyocyte proliferation. Meanwhile, lncRNA CPR deficiency can accelerate cardiomyocyte proliferation, promote myocardial repair and improve cardiac function after myocardial infarction (Ponnusamy et al., 2019).

Besides, lncRNAs can binding to target DNA as a DNA-RNA heteroduplex, DNA-DNA-RNA triple strand (Hung and Chang, 2010). lncRNA *Sarrah* is significantly down-regulated in aging and infarct hearts, regulates NRF2 gene transcription by recruiting gene promoters CRIP2 and p300 to form a DNA-DNA-RNA triple helical structure. Silencing *Sarrah* can induce apoptosis of cardiomyocytes, conversely, an increase of *Sarrah in vivo* can improve cardiomyocyte survival rate and myocardial systolic function after AMI (Trembinski et al., 2020).

### Signal

Under different stimulus conditions, lncRNA will be specifically transcribed and participate in the conduction of special signaling pathways as signal transduction molecules. Signal lncRNAs mediate gene expression through *cis-* or *trans-* action. In cis, lncRNAs can regulate the expression of adjacent genes, enhancer lncRNAs synthesized at enhancers can activate adjacent genes *cis* through DNA loops. In trans, it can affect the expression of genes across chromosomes (Batista and Chang, 2013). Although some lncRNAs are *cis-*regulators (Koerner et al., 2009), recently, loss of function tests has verified that most lncRNAs have *trans-*regulatory effects, such as knockout of some lincRNAs with no effect on adjacent genes or low effect on other genes (Ørom et al., 2010; Guttman et al., 2011).

After being transcribed, some lncRNA molecules have the function of regulating downstream gene transcription. The expression of lncRNA FAF is downregulated in ischemia-hypoxic cardiomyocytes and heart tissues of AMI rats. lncRNA FAF regulates the expression of FGF9/FGFR2 in myocardial cells in the form of positive feedback, then inhibits apoptosis by affecting PI3K/Akt signaling pathway (Shi et al., 2019). Microarray analysis showed that lncRNA SLC8A1-AS1 was down-regulated in AMI, prevented myocardial injury by down-regulating SLC8A1 and activating the cGMP-PKG signaling pathway, inhibiting the release of pro-inflammatory factors and reducing the infarct size to alleviate myocardial ischemia damage (Guo et al., 2019). lncRNA antisense Igf2r RNA (Airn, also known as “Air”) is an imprinted gene transcribed and expressed from the parent chromosome. Its transcription is necessary to inhibit several imprinted genes on the parent chromosome in a tissue-specific and allele-specific manner. Its transcription plays a key role in overlapping gene silencing (Wang and Chang, 2011). In addition to its function as an imprinting gene, Airn can post-transcriptionally control protein-coding genes in cardiomyocytes. In HL-1 cells treated with H_2_O_2_, Airn binds to insulin-like growth factor 2 mRNA-binding protein 2 (Igf2bp2) and affects the translation of Igf2bp2, thereby controlling the downstream mRNA. Silencing Airn can increase apoptosis and affect the physiological function of cardiomyocytes (Hosen et al., 2018). It indicates that Airn belongs to translation-modulating lncRNA, which can modify protein function by directly interacting with the protein itself or binding partners.

ZnFX1 antisense RNA 1 (ZFAS1) belongs to lncRNA and has been reported to promote the occurrence and development of a variety of cancers. However, a series of studies on ZFAS1 in acute myocardial infarction (AMI) has shown that ZFAS1 is significantly differentially expressed between AMI patients and healthy people. The circulation level of ZFAS1 can be used as an independent predictor of AMI and is considered a novel biomarker. Besides, the combination of ZFAS1 with lncRNA CDR1As could increase the power of sensitivity and specificity of the prediction of AMI (Zhang et al., 2016). The expression of ZFAS1 was also significantly increased in the cytoplasm and sarcoplasmic reticulum of the MI mice model and hypoxic cell model. At the subcellular level, ZFAS1 causes Ca^2+^ overload in cardiomyocytes and induces mitochondrial swelling and a decrease in mitochondrial membrane potential. At the molecular level, multiple sequence fragments of ZFAS1 were found to be complementary to the 3′-UTR of SERCA2a (sarcoplasmic reticulum Ca^2+^-ATPase 2a), limiting its activity and expression, and activating apoptotic pathways. These results suggest that ZFAS1 may interact with SERCA2a mRNA through direct antisense complementation to induce the degradation of SERCA2a mRNA and change the expression level (Zhang et al., 2018; Jiao et al., 2019). Thus, ZFAS1 may be an endogenous SERCA2a inhibitor that inhibits its function by restricting its intracellular protein-bind SERCA2a levels.

### Scaffold

Some lncRNAs compose scaffolds through specific secondary structures and bind to different protein molecules to form complexes. These complexes are involved in *cis* or *trans* epigenetic regulation of genes as transcriptional activators or inhibitors (Nagano and Fraser, 2011; Guttman and Rinn, 2012). The association between lncRNA and disease may be related to scaffold capacity (Ribeiro et al., 2018). For example, lncRNA can affect mRNA splicing, translation, or degradation by binding to mRNA or protein components of the RNP complex (Gong and Maquat, 2011; Rinn and Chang, 2012; Batista and Chang, 2013).

Expression of lncRNA *Ahit* was upregulated in the hearts of mice with aortic contraction. Both *in vivo* and *in vitro* experiments confirmed that *Ahit* binds and recruited SUZ12, the core protein of PRC2, to the promoter of MEF2A, acting as a scaffold and initiates H3K27me3 to mediate the downregulation of MEF2A, thereby regulating cardiac hypertrophy. Meanwhile, clinical data confirmed that *Ahit* was significantly elevated in serum samples from patients with hypertensive heart disease. These results indicate that *Ahit* is a kind of lncRNA that plays an important scaffold role through epigenomic regulation, modulates chromatin remodeling in the dense state (Yu J. et al., 2020). Coronary atherosclerotic heart disease (CAD) often causes myocardial ischemia and myocardial infarction, leading to sudden cardiac death. The research showed that the expression of ANRIL in CAD patients was significantly higher than that in healthy people. As the scaffold, ANRIL could promote the binding of WDR5 and HDAC3 to form protein complexes that regulate the expression of target genes through histone modification, up-regulate ROS level and promote the phenotypic transformation of the human aortic smooth muscle cell, which lead to the progression of CAD (Zhang et al., 2020).

## Exosome

In multicellular organisms, cells exchange information with neighboring or distant cells by secreting individual molecules or extracellular vesicles (EVs) of intracellular origin, including exosomes, microvesicles (Tkach and Théry, 2016; Barile et al., 2017). Exosomes are the smallest subset of EVs with a diameter ranging from 30 to 150 nm. Unlike other EVs, exosomes have special membranous surface molecules that allow them to target the recipient cell and release their contents into the cell, thereby altering the state of the recipient cell (Tkach and Théry, 2016). Exosomes carry a variety of molecules, including low molecular compounds, lipids, proteins, and various nucleic acids, such as DNA, mRNA, and ncRNA (Valadi et al., 2007). The composition of exosomes varied greatly in different cell types and microenvironments, even exosomes released by the same type of cells under healthy and pathogenic conditions are also different. Therefore, exosomes can be used as both diagnostic markers and therapeutic targets for diseases.

Exosomes are released by a variety of heart cells, including cardiomyocytes, endothelial cells, cardiac fibroblasts, and cardiac progenitor cells (Barile et al., 2017). Hypoxia is an effective exosomal-releasing stimulator in cardiomyocytes, inducing the heart to release EVs containing antiapoptotic proteins (e.g., pro-apoptotic and anti-apoptotic factors) (Chistiakov et al., 2016). During AMI, the damaged cardiomyocytes produce exosomes with angiogenesis, anti-apoptosis, mitosis, and increased growth factor content to induce repair and healing of the infarct myocardium. RNA-seq analysis showed that lncRNA AK139128 was overexpressed from both hypoxic cardiomyocytes and its exosomes. Interestingly, hypoxic exosomes increased the expression of AK139128 in recipient cardiac fibroblasts (CFs) after co-culture with CFs. Besides, exosome AK139128 from hypoxic CMs can stimulate CFs apoptosis and inhibit proliferation (Wang and Zhang, 2020).

Some studies have confirmed lncRNA-UCA1 might participate in the process of ischemia and hypoxia of cardiomyocytes (Wang Q.S. et al., 2020). Exosome lncRNA-UCA1 deriving from hypoxia mesenchymal stem cells (hMSCs) plays a cardioprotective role through the miR-873-5p/XIAP axis. Different from healthy people, the plasma of AMI patients contains exosomes rich in lncRNA-UCA1, and circulating exosomes lncRNA-UCA1 may be a promising new biomarker for the diagnosis of AMI (Sun et al., 2020). Another lncRNA contained in hMSCs exosomes is KLF3-AS1. *In vivo* transfection of KLF3-AS1 exosomes in rats, and *in vitro* incubation of hypoxic cardiomyocytes, also KLF3-AS1 exosomes, both confirmed that overexpression of KLF3-AS1 resulted in the reduction of myocardial infarction area and apoptosis, and KLF3-AS1 acted as an RNA sponge-miR-138-5p to mediate the expression of SIRT1, thereby regulating cardiomyocyte apoptosis and progression of myocardial infarction (Mao et al., 2019).

Previous studies have demonstrated that atorvastatin (ATV) pretreatment can enhance the efficacy of mesenchymal stem cells (MSCs) in the therapeutic of AMI (Yang et al., 2008), also, the combination therapy of ATV and MSCs can significantly improve cardiac function after AMI (Yang et al., 2009). Exosomes isolated from ATV-pretreated mesenchymal stem cells (MSC^ATV^-Exo) increased cell survival and angiogenesis, improved cardiac function, and reduced infarct size and myocardial cell apoptosis, both *in vitro* and *in vivo*. Molecular mechanism studies have shown that lncRNA H19 is a mediating role of MSC^ATV^-Exo in regulating miR-675 expression and the activation of pro-angiogenic factor VEGF and intercellular adhesion molecule-1 (ICAM-1). Meanwhile, the downregulation of lncRNA H19 decreased the cardioprotective effect of MSC^ATV^-Exo. These results suggested that lncRNA H19 mediated the cardioprotective effect of MSC-derived exosome in promoting angiogenesis and cardioprotective effects in myocardial infarction (Huang P. et al., 2020).

A large number of studies have shown that ncRNAs contained in exosomes secreted by stem cells or somatic cells can serve as biomarkers for myocardial infarction, as well as myocardial protection and myocardial regeneration after infarction injury (Khan et al., 2015; Gallet et al., 2017). Circulating exosomal lncRNAs ENST00000556899.1 and ENST00000575985.1 were significantly up-regulated in AMI patients compared with healthy people, they showed related with clinical parameters, including inflammatory biomarkers, prognostic indicators, and myocardial damages markers, functioning as potential biomarkers for predicting the prognosis of patients with AMI (Zheng et al., 2020).

Exosomes have lipid bilayer, compared with the lncRNAs in extracellular fluid, the lncRNAs in packaged exosomes are highly stable because of the protection from enzymatic degradation, which may have long-term stable existence in the cardiovascular system (Cheng et al., 2019). Therefore, exosomes lncRNA could be a novel biomarker and therapeutic approach (Table 2).

**TABLE 2:** Exosomes lncRNAs of myocardial infarction.

LncRNA	Target	Function	References
UCA1	miR-873-5p/XIAP	Improve the level of antiapoptotic protein and cardiac protection	Sun et al., 2020
KLF3-AS1	miR-138-5p/SIRT1	Inhibit cell pyroptosis and attenuate MI progression.	Mao et al., 2019
H19	miR-675/VEGF/ICAM-1	Mediate the cardioprotective effect of MSC-derived exosomes and promoted the angiogenesis of MI.	Huang P. et al., 2020

## lncRNAs and Secondary Changes of MI

### Ischemia and Reperfusion

Reducing myocardial infarct size and preserving the left ventricular systolic function is necessary for STEMI (ST-segment elevated myocardial infarction) patients. The most effective therapeutic strategies used in clinical practice include primary percutaneous coronary intervention (PPCI) and drug intervention (Hausenloy, 2012). However, the process of restoring coronary blood flow to the coronary artery to ischemic tissue may induce cardiomyocyte death and myocardial injury, a phenomenon termed “myocardial reperfusion injury” (Yellon and Hausenloy, 2007). Previous studies on rodent ischemia-reperfusion (I/R) models have shown that I/R leads to dysregulation of lncRNA expression in heart tissues, and these dysregulated lncRNAs are associated with downstream molecular mechanisms of ischemia-reperfusion (Lin et al., 2018; Lou et al., 2021).

lncCIRBIL, lncRNA Cardiac Injury-Related Bclaf1-Inhibiting LncRNA, reduced in ischemia-reperfusion heart tissue, and lncCIRBIL knockout mice aggravated cardiac I/R damage. Studies have shown that lncCIRBIL is located in the cytoplasm of cardiomyocytes and can directly bind to Bclaf1 (Bcl2-associated transcription factor 1) to inhibit its nuclear translocation to activate the p53 transcriptional activity, which is a necessary step leading to cardiac I/R injury. Therefore, lncCIRBIL and Bclaf1 are key regulators of cardiac I/R injury and potential therapeutic targets for ischemic heart disease. This is a study on the interaction between lncRNA and protein as the mechanism of myocardial ischemia-reperfusion (Zhang Y. et al., 2021). During myocardial ischemia-reperfusion, there are also ceRNA mechanisms. Endogenous lncRNA HOTAIR is an important negative regulator of oxidative stress and cardiomyocyte apoptosis in myocardial I/R injury. HOTAIR can prevent oxidative stress, cardiomyocyte apoptosis, and cardiac dysfunction by regulating the Zeste homology 2/microRNA-451/calcium-binding protein 39 (EZH2/miR-451/CAB39) axis enhancer to activate AMPKα (Meng et al., 2020).

### Myocardial Fibrosis

After myocardial infarction, myocardial fibrosis is a characteristic and inevitable event. Cardiac fibroblasts secrete excessive extracellular matrix (ECM), which eventually leads to collagenous scar replacing the dead ischemia myocardium (Frangogiannis, 2012). The generation and maintenance of scars is the key to prevent the heart from infarct area expanding. But excessive deposition of the ECM leads to heart stiffness, which can eventually lead to heart failure and death (Travers et al., 2016; Frangogiannis, 2017). lncRNA H19 was significantly upregulated in the infarct area and reaches an exceptionally high level at post-MI day 4, then competes with COL1A1 to form the H19-YB-1 complex under hypoxia, resulting in decreased COL1A1 expression, thus preventing ECM deposition and cardiac remodeling (Choong et al., 2019).

Another study reveals a novel function and molecular mechanism of lncRNA in myocardial fibrosis induced by myocardial infarction. lncRNA 554 was highly expressed in cardiac fibroblasts after myocardial infarction for 28 days. *In vitro*, knockout of lncRNA 554 significantly reduced in CFs migration and ECM expression. *In vivo*, knockout of lncRNA 554 inhibited myocardial fibrosis and improved myocardial function. TGF-β1 inhibitor (TEW-7197) could significantly inhibit the fibrosis-promoting function of lncRNA 554 in CFs. These results indicated that the effect of lncRNA 554 on MI-induced myocardial fibrosis was dependent on the TGF-β1 signaling pathway (Luo et al., 2020). Wisper (WISP2 super enhancement factor associated RNA) is a myocardial fibroblast enriched lncRNA that regulates myocardial fibrosis. The expression level was correlated with the degree of myocardial fibrosis in mouse models of myocardial infarction and aortic stenosis patients. Functionally, Wisper modulates cardiac fibroblast gene expression programs that are critical for cellular characterization, extracellular matrix deposition, proliferation, and survival (Micheletti et al., 2017).

### Heart Failure

Heart failure (HF) is a common disease after AMI (Velagaleti et al., 2008; Ezekowitz et al., 2009), and the incidence of HF increases significantly after MI and is associated with mortality (Lewis et al., 2003). Studies have shown that lncRNAs are differentially expressed in various HF models in mice and humans. After pressure overload, which is induced by aortic contraction, the left ventricular whole gene of heart failure mice was changed, among which 135 lncRNAs were expressed in the left ventricle of heart failure mice (Lee et al., 2011).

The expression level of lncRNA in the human HF tissues was also changed. A study of 40 human LV samples through next-generation sequencing revealed significant differences in mRNA, miRNA, and lncRNA expression patterns between ischemic and non-ischemic failure hearts, most of these lncRNAs are encoded by mitochondrial DNA. In this study, the authors found that expression patterns of lncRNAs, not mRNAs or miRNAs, are more sensitive in the discrimination of ischemic and non-ischemic heart failure (Yang et al., 2014). The above results indicate that lncRNA can be a good biomarker in heart disease states. Besides, this study also found a strong positive correlation between lncRNA expression and its adjacent genes, suggesting that there may be a *cis-*regulated transcription mechanism in heart failure myocardium.

### Cardiac Remodeling

Loss of myocardial function is a pathological feature of AMI, the injured heart undergoes structural and functional changes to maintain cardiac outcome, such changes in the heart are term as cardiac remodeling. Since cardiac remodeling affects ventricular function and is associated with the patient’s prognosis (Bostan et al., 2020), the phenomenon of ventricular remodeling in MI patients has attracted more and more attention. Studies have shown that ventricular remodeling in the infarct area and adjacent areas lead to the loss of shortening and contraction with dyssynchronization, resulting in decreased cardiac output, as well as the increase in ventricular volumes (van den Borne et al., 2010).

lncRNA Chast (cardiac hypertrophy-associated transcript) was up-regulated in hypertrophic heart tissue and transverse aortic constriction-operated mice. Chast suppresses Pleckstrin homology domain-containing protein family M member 1 (Plekhm1) then prevents cardiac remodeling and cardiomyocyte autophagy (Viereck et al., 2016). Among the 20 upregulated lncRNAs in MI mice, the myocardial infarct-associated transcript 1 (MIRT1) and MIRT2 were significantly upregulated. In the time-process analysis, the expressions of MIRT1 and MIRT2 progressive increased after MI and peaked 24 h, then returned to baseline 2 days later. On the other hand, MIRT1 and MIRT2 levels correlated with the expression of multiple left ventricular remodeling genes. Expression of MIRT1 and MIRT2 displayed progressive increases after induction of MI, suggesting that MIRT1 and MIRT2 may contribute to left ventricular remodeling after myocardial infarction (Zangrando et al., 2014).

Details of lncRNAs modes of functions in MI-induced secondary changes are shown in Table 3.

**TABLE 3:** LncRNAs in secondary changes of myocardial infarction.

LncRNA	Target	Function	References
lncCIRBIL	Bclaf1	Reduced infarcted area after I/R and cardiac protection.	Zhang Y. et al., 2021
HOTAIR	EZH2/miR-451/CAB39	Regulates oxidative stress and cardiac myocardial apoptosis during I/R	Meng et al., 2020
H19	COL1A1	Form H19-YB-1 complex to decrease COL1A1 expression and preventing ECM deposition and cardiac remodeling after MI.	Choong et al., 2019
SAIL	SAFB	Regulating cardiac fibrosis by promoting SAFB bind to rpb1.	Luo et al., 2021
*Safe*	*Sfrp2* mRNA	Binding *Sfrp2* mRNA 3′-UTR and inhibited TGF-β- induced fibrosis.	Hao et al., 2019
*Cfast*	COTL1/TRAP1/TGF-β signaling pathway	Competitively inhibits the interaction between COTL1 and TRAP1, thereby enhancing fibrosis-related gene expression and myofibroblasts transdifferentiating into CFs.	Zhang F. et al., 2021
lncRNA 554	TGF-β1 signaling pathway	Regulate CFs migration and ECM expression following MI.	Luo et al., 2020
Chast	Plekhm1	Prevent cardiac remodeling and hypertrophy.	Viereck et al., 2016
Wisper	TIAR	Reduce the development of myocardial fibrosis after MI and prevent adverse remodeling.	Micheletti et al., 2017

## lncRNAs and Roles as Novel Biomarkers of MI

The European Society of Cardiology (ESC) and the American College of Cardiology (ACC) have introduced sensitive cardiac biomarkers to the definition of MI by biochemical and clinical methods (Alpert et al., 2000). Cardiac troponin I (cTnI) and T (cTnT) are preferred biomarkers for the assessment of myocardial injury and can be defined as a specific subtype of MI (Thygesen et al., 2018). High sensitivity-cTn assays have been widely used in nowadays routine assays. The definition of myocardial infarction, published in 2018 by the Journal of the American Heart Association, states that myocardial infarction is the presence of acute myocardial injury with the rise and/or fall of cTn with at least one value above the 99th percentile upper reference limit (URL) in the context of the evidence of acute myocardial ischemia (Thygesen et al., 2018). cTns have guided treatment decisions for CVD for decades. However, the increased sensitivity of hs-cTn (high-sensitivity cardiac troponin) also increases the likelihood of false positives in a healthy population (Schulte et al., 2020), so there is a need to improve and supplement existing biomarkers, with circulating lncRNA potentially contributing to the specificity of protein biomarkers.

A study tested five lncRNAs (aHIF, ANRIL, KCNQ1OT1, MIAT, MALAT1) that associated with cardiac pathology in the blood samples from MI patients, results shows that the levels of certain lncRNAs in peripheral blood cells are regulated after MI and associated with LV dysfunction (Vausort et al., 2014). Levels of aHIF, KCNQ1OT1, and MALAT1 were higher in MI patients than in healthy volunteers (*P* < 0.01), and levels of ANRIL were lower in patients with MI (*P* = 0.003). Interestingly, Levels of ANRIL were associated with cardiovascular risk factors, such as age, diabetes mellitus, and hypertension. In multivariable and reclassification analyses, ANRIL and KCNQ1OT1 improved the prediction of left ventricular dysfunction by a model, including demographic features, clinical parameters, and cardiac biomarkers.

A genome-wide association study of MI patients in Iceland has found an association between MI and common sequence variations on chromosome 9p21. Carriers were 1.64 times more likely to suffering MI than non-carriers, and 2.02 times more likely to develop MI in early-stage cases (Helgadottir et al., 2007). The strongest genetic susceptibility locus for coronary artery disease is located in the chromosome 9p21 gene desert (Schonrock et al., 2012). The *INK4* locus is located on human chromosome 9p21 and encodes proteins called p16INK4a, ARF, and P15INK4B (Gil and Peters, 2006). It was found that antisense non-coding RNA (ANRIL), a long-stranded non-coding RNA, transcribed from the INK4 site could bind and recruit PRC1 and PRC2 to the INK4 locus, thus inhibiting the transcription of p16INK4A and p15INK4B (Kotake et al., 2011). p16INK4a, ARF, and P15INK4B play a key role in regulating cell proliferation, cell aging, and apoptosis (Kim and Sharpless, 2006). These changes are important features of atherosclerosis and are the root cause of myocardial infarction and CAD (Minamino and Komuro, 2007). Therefore, it is worth noting that this variant, in addition to affecting MI, may also increase the risk of CAD.

According to research, in the plasma of patients at the early and late stage of myocardial infarction, seven lncRNAs were differentially expressed during left ventricular remodeling. And the expression of LIPCAR, a mitochondrial source long intergenomic non-coding RNA, is associated with cardiac remodeling, and the elevation of LIPCAR levels in patients with HF may be significantly associated with a higher risk of mortality, which can be used to independently predict cardiovascular mortality in patients with chronic heart failure (Kumarswamy et al., 2014). Another research data confirmed that circulating long non-coding RNA cardiac hypertrophy-associated transcript (CHAST) was an independent predictor of myocardial systolic function in patients with early AMI (*P* < 0.05), and CHAST could be used as a candidate biomarker for cardiac remodeling. Compared with the control group, CHAST level was higher in patients with AMI, and the expression level of CHAST was positively correlated with cardiac systolic function at 24 h (*P* < 0.05) (Wang X. et al., 2020).

The characteristics of an ideal biomarker include four aspects: high sensitivity, high specificity, easy detection, and minimal invasiveness (Bostan et al., 2020). High sensitivity refers to increased concentration after the onset of disease, rapid release, and long half-life to allow diagnosis. High specificity means that it is specifically expressed in cardiac tissue and is not present in healthy people’s hearts. Also, the ideal biomarker needs to be easy to detect, effective in clinical diagnosis and treatment and can help predict prognosis (Bostan et al., 2020). The ideal biomarker should satisfy multiple characteristics making it difficult to identify a single parameter that satisfies all of these; therefore, the combination of biomarker analysis in clinical diagnosis can improve the accuracy, while lncRNA as a biomarker still needs more accurate detection and verification.

## lncRNAs and Therapeutic Perspective

A large number of studies have shown that abnormal expression of lncRNA can lead to cardiovascular diseases, and heart-specific gene regulation shows great potential in preventing and alleviating heart diseases, indicating that lncRNA is a new target with diagnostic efficacy and therapeutic potential. Unlike protein, lncRNAs are easy to synthesis and delivery, restoring abnormal lncRNA expression in the disease through gene therapy may be a new therapeutic strategy. RNA-based therapy is divided into RNA molecules used as therapeutic drugs (Crooke et al., 2018) and RNA-targeted small-molecule drugs (Warner et al., 2018). Cytoplasmic lncRNAs can be downregulated by siRNA or shRNA. For nuclear lncRNAs, excessive expression of lncRNA can be inhibited by antisense oligonucleotide to inhibit inappropriate upregulation (Rincon et al., 2015).

### Adeno-Associated Virus Vectors

Adeno-associated virus vectors (AAV) are single-stranded DNA vectors capable of persistent transgenic expression in target tissues, including the heart (Levy et al., 2020). The use of lncRNA-CAREL transgenic mice, as well as adenovirus-mediated endogenous CAREL silencing *in vivo*, all could significantly promote cardiac regeneration and improved cardiac function after myocardial infarction (Cai et al., 2018). Adenovirus-mediated silencing of endogenous lncDACH1 promotes cardiac regeneration in MI mice and activates the proliferative potential of cardiomyocytes, which provides a novel therapeutic strategy for ischemic heart disease (Cai et al., 2020). AAV9 is the most effective serotype of cardiac gene transfer, and some mouse model studies have reported the superiority in cardiac gene transfer (Pacak et al., 2006). The recombinant AAV9 vector carrying the lncRNA Oip5-as1 promoter (AAV9-Oip5-as1) was injected into the myocardial tissue of MI rats for gene transfer therapy. The results showed that AAV9-Oip5-as1 injection effectively increased Oip5-as1 expression in the heart of MI rats, which significantly inhibited the increase in myocardial infarction size (Niu et al., 2020). These results suggest that lncRNA can regulate the proliferation of cardiomyocytes and postnatal heart development. Targeting lncRNA may protect the heart after myocardial infarction by inducing the replication of myocardial cells, which may be considered as a new therapeutic method to promote the repair of the damaged heart (Thum, 2018). These results indicate that targeting lncRNA may protect the heart after myocardial infarction, and AAV targeting therapy of heart-targeted gene delivery is effective, which may be considered as a new therapeutic approach to promote damaged heart repair.

### Antisense Oligonucleotides

One of the advantages of oligonucleotide drugs is to upregulate specific target genes or proteins, which is difficult to do with small molecule drugs before. Antisense oligonucleotides (ASO) delivered into cells through complementary base pairs, target intracellular mRNAs or functional ncRNAs, and treat diseases by leading to gene silencing or controlling gene expression through different mechanisms, including inhibition of the activity of natural antisense transcripts, interaction with ncRNA promoter binding sites, and blocking of regulatory and/or miRNA binding sites in the 3-′UTR (Yu A.M. et al., 2020). The antisense oligonucleotide method has been successfully applied in clinical practice, but the application of anti-lncRNA in the cardiovascular field is still in the pre-clinical stage. Since lncRNA have multiple target genes and target proteins, these antisense sequences must be chemically modified to improve the structural stability and tissue distribution of nucleotides (Landmesser et al., 2020).

With the invention of genome-editing techniques such as CRISPR/Cas9, it is possible to generate and identify RNA mutants. However, the artificial phenotype caused by the CRISPR off-target effect still needs to be overcome. While most of the current research is still carried out at the cellular level or in small rodent model systems, research in large mammal studies or human *ex vivo* studies needs to be advanced quickly to facilitate the future clinical success of the next generation of ncRNA-based therapies (Huang C.K. et al., 2020). Besides, there is growing evidence that extracellular vesicles may be a very promising area of research for targeted cardiac delivery (Sahoo et al., 2021).

### lncRNAs Regulating Cell Cycle Progression

Exogenous delivery of lncRNAs can inhibit cell death and restore cardiac function after myocardial infarction. In addition, some studies have shown that lncRNAs also could regulate cardiomyocyte proliferation by enhancing or inhibiting cell cycle processes. Comprehensive analysis of transcriptome changes from human fetal-to-adult heart transition found that differentially expressed lncRNAs may be potential targets involved in cardiac regeneration after MI.

For example, the lncRNA CPR mentioned above is significantly higher in the adult heart than in the fetal stage. CPR inhibits MCM3 expression by directly interacting and recruiting DNMT3A to CpG islands of MCM3 promoter region, thereby prevent cardiomyocyte proliferation. The results show that CPR is a negative regulator of cardiomyocyte proliferation in postnatal and adult hearts (Ponnusamy et al., 2019). On the contrary, lncRNA ECRAR (Endogenous Cardiac Regeneration-associated Regulator) may serve as an effective gene target for heart (Chen et al., 2019). ECRAR promotes myocardial regeneration in postnatal and adult rat hearts and reduces adverse remodeling after myocardial infarction. ECRAR was transcriptionally upregulated by E2F1, and then directly bound to and promoted the phosphorylation of ERK1/2, resulting in downstream activation of cyclin D1 and cyclin E1 activation, which in turn activated E2F1. The E2F1-ECRAR-ERK1/2 formed a positive feedback loop to drive cell cycle progression and promoted CM proliferation after myocardial infarction.

## Discussion

### lncRNAs and Current Challenges

The discovery of non-coding RNAs, such as lncRNAs, have given valuable insights into the molecular mechanisms of cardiovascular diseases such as myocardial infarction, also provides us with a new understanding of the pathogenesis of MI. But there are still many obstacles to overcome before lncRNAs can be used in clinical practice.

Firstly, considering the versatility of lncRNA in the pathophysiological process of the human body, off-target hits of interactions may lead to adverse effects, so a major problem with the use of lncRNAs in therapy is the highly cardio-specific targeted drug delivery systems. Although lncRNA is low conserved in sequence, it has high tissue specificity. According to unsupervised cluster analysis and RNA-seq analysis, the majority of lncRNAs have a tissue-specific expression (Cabili et al., 2011). Therefore, with the development of oligonucleotide delivery and gene therapy, we thought it is necessary to find highly specific and efficient targeting delivery systems to regulate the target lncRNA.

Secondly, although many studies have provided many potential therapeutic targets of lncRNAs and achieved success in animal models, these findings are not necessarily transferable to humans. Besides, most of the cardiac animal model are mice or rats, but rodent heart is significantly smaller than that of humans. It has been reported that the efficiency of gene transfer is usually inversely proportional to the size of the host (Ylä-Herttuala and Alitalo, 2003), so the animal heart cannot properly mimic the disease and treatment of the human heart. Therefore, the clinical transformation of lncRNAs still faces challenges. We believe that in future research, more effective methods are needed to study the specific functions and safety of lncRNAs in lab trials, and animal models that are more similar to human diseases are also needed for clinical transformation.

Thirdly, as an epigenetic regulatory factor, lncRNAs play a crucial role in gene regulation and disease pathogenesis, but their detailed molecular mechanisms have not been fully elucidated. The function of lncRNAs may be closely related to secondary structure due to a low level of sequence conservation and protein-coding ability. The biological functions of the lncRNAs require further investigation and deep study to provide a better understanding of the molecular structure in MI. In addition, the further development of lncRNA-targeted therapy depends on in-depth research on lncRNA, detailed structure and functional studies are more conducive to targeted and intervention design. Therefore, further research of the genomic and subcellular localization of lncRNAs, and their interaction relationships with proteins and other nucleic acids will provide additional insights into the understanding of mechanisms and functions, in which the expression level of lncRNAs are expected to be modulated in a tissue- and cell-specific manner.

Finally, the authenticity of lncRNA in myocardial infarction still needs more extensive verification before being considered as a true regulator of MI pathogenesis. Although many studies have found differentially expressed lncRNAs in MI, extensive verifications are still needed. Because an uncontrollable factor is a difference in type and number of cells in the infarct tissue, this may lead to false positives. Besides, different platforms and technologies may result in inconsistent results. Therefore, to obtain the most stable variational and reliable heart-specific lncRNAs in MI, it is necessary to record the whole transcriptome information of expression in specific cardiac regions at different developmental stages of myocardial ischemia or infarction.

### lncRNAs and Future Prospects

lncRNAs participate in all aspects of physiology and pathology of the human body, and have been involved in cardiovascular disease, cancer, metabolism, and immunology. They play a role through molecular and biochemical mechanisms, from *cis-* to *trans-* regulation of gene expression, from epigenetic regulation of nucleus to post-transcriptional control of cytoplasm. Although we classify lncRNAs into four archetypes of molecular mechanisms above, some lncRNAs have the biological function of integrating multiple archetypes, and can control each level of multi-level regulation of gene expression pathway (Wapinski and Chang, 2011), which play a wide range of regulatory roles in almost every stage of gene expression. Besides, individual lncRNA can regulate several downstream genes, and one functional gene can be targeted by multiple lncRNAs.

The biology of lncRNA is a rapidly developing field in cardiovascular research, although some preliminary advances have been made, it is undeniable that the study of lncRNAs is still in its infancy. Further research is still needed to fully translate existing basic research findings into clinical treatment. Therefore, we believe that in future research on the mechanism and treatment of myocardial infarction, we need to further understand the lncRNA localization and structure, the interaction between proteins and nucleic acids, and the specific physiological function of lncRNAs in animal models. In addition, we also believe that it is necessary to consider the combination of multiple treatment methods of MI therapeutics strategies.

## Conclusion

In summary, this review mainly reviews the regulatory gene networks of lncRNAs in cardiac development and pathology progress of myocardial infarction, and the secondary changes caused by myocardial infarction are also involved. Monitoring the quality and quantity of lncRNAs after myocardial infarction may become a new factor for the evaluation and prediction of prognosis, and lncRNAs may also become a new therapeutic target after myocardial infarction, providing a new idea for treatment. The abundance and diversity of differentially expressed lncRNA transcripts in diseases provide the possibility of diagnosis and treatment at the RNA level, but it also poses great challenges.

## Author Contributions

LX conceived the conception and wrote the manuscript. LL provided a lot of valuable advice and contributed to the revision. JM, QZ, and JZ provided many constructive suggestions and discussions. All authors read and approved the final manuscript.

## Conflict of Interest

The authors declare that the research was conducted in the absence of any commercial or financial relationships that could be construed as a potential conflict of interest.

## Publisher’s Note

All claims expressed in this article are solely those of the authors and do not necessarily represent those of their affiliated organizations, or those of the publisher, the editors and the reviewers. Any product that may be evaluated in this article, or claim that may be made by its manufacturer, is not guaranteed or endorsed by the publisher.

## Footnotes

1
http://www.noncode.org
